# Atypical localization of demodicosis after COVID‐19 infection

**DOI:** 10.1002/ccr3.5446

**Published:** 2022-02-13

**Authors:** Ines Lahouel, Randa Said El Mabrouk, Rim Hadhri, Monia Youssef, Hichem Belhadjali, Jameleddin Zili

**Affiliations:** ^1^ Dermatology department Fattouma Bourguiba Hospital University of Medicine Monastir Tunisia; ^2^ Dermo‐respiratory research laboratory University of Medicine Monastir Tunisia; ^3^ Anatomo‐pathology department Fattouma Bourguiba Hospital University of Medicine Monastir Tunisia

**Keywords:** COVID‐19, demodicosis, dermatology, immunodepression, parasitology, scalp

## Abstract

Since its outbreak in December 2019, a consistent number of case reports have been published describing a complex spectrum of skin manifestations associated with COVID‐19. We report a first observation of demodicosis of the scalp after a severe acute respiratory syndrome coronavirus 2 (SARS‐COV‐2) infection.

## 1 CASE PRESENTATION

**FIGURE 1:**
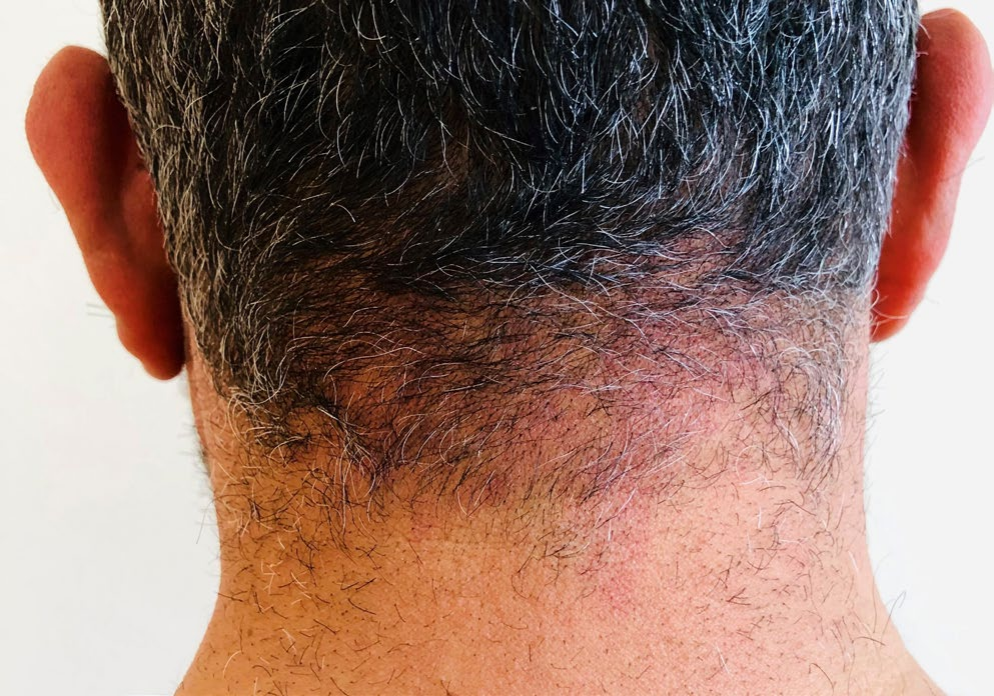
Diffuse erythema with erythematous papules associated with hair thinning in the occipital scalp

**FIGURE 2:**
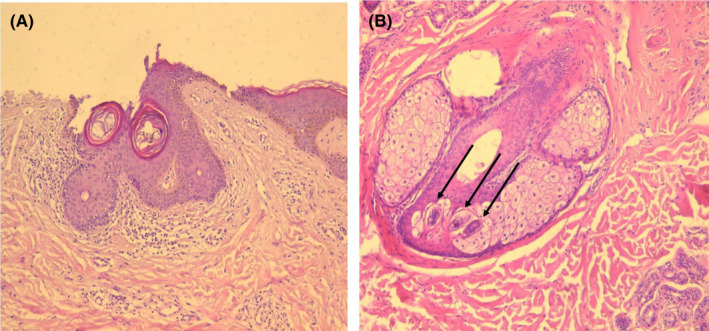
(A) Lymphocytic infiltrate around follicles (hematoxylin‐eosin (100×)). (B) Multiple Demodex mites within pilosebaceous follicle (hematoxylin‐eosin (400×))

A 45‐year‐old male patient presented with a lesion of the scalp appearing 10 days after a moderate SARS‐CoV‐2 infection. The skin examination found many firm and erythematous papules on a background of a diffuse erythema associated with hair thinning in the occipital region (Figure 1). Skin biopsy revealed excessive Demodex mites in the follicular infundibulum (Figure 2B) with perivascular and perifollicular lymphocytic infiltration (Figure 2A). Based on the clinical and histopathological data, the diagnosis of scalp demodicosis was retained. The patient was treated with topical metronidazole. A complete regression of the lesions was obtained in two weeks.

## 2 DISCUSSION

Demodicosis is an ectoparasitosis caused by the proliferation of a mite: *Demodex sp*, which permanently resides in or near the pilosebaceous unit and seborrheic glands. There are two types of demodicosis: Primary demodicosis includes pityriasis folliculorum, papulopustular, and ocular and auricular demodicosis. Secondary demodicosis is usually associated with systemic or local immunosuppression.^1^ Scalp demodicosis is rare and is described mainly in patients with weakened immune systems.^2^ Immunodeficiency appears to create a favorable environment for the development of the parasite. Our patient presents probably a secondary form of demodicosis, which can be explained by the disturbances of the host's immune response caused by SARS‐CoV‐2 infection.

## CONFLICT OF INTEREST

None.

## AUTHOR CONTRIBUTIONS

Ines Lahouel: wrote the manuscript. Randa Said El Mabrouk: wrote the manuscript and submitted the revised article. Rim Hadhri: wrote the histological part of the manuscript. Monia Youssef: supervised and approved the revised manuscript. Hichem Belhadjali: supervised and approved the revised manuscript. Jameleddin Zili: supervised and approved the revised manuscript. Consent statement: approved by all authors.

## ETHICAL APPROVAL

Ethics statements for this article were approved.

## CONSENT

Written consent was obtained from the patient to publish this report in accordance with the journal's patient consent policy.

## Data Availability

Data sharing is not applicable to this article as no new data were created or analyzed in this study.
